# Integrating molecular genetics into thyroid cancer staging: A critical perspective on personalized risk stratification in thyroidology

**DOI:** 10.1016/j.clinsp.2026.101000

**Published:** 2026-05-28

**Authors:** Ilker Sengul, Demet Sengul

**Affiliations:** a Thyroidology Unit, Giresun University Faculty of Medicine, TR28100 Giresun, Turkey; b Division of Endocrine Surgery, Giresun University Faculty of Medicine, Giresun, Turkey; c Department of General Surgery, Giresun University Faculty of Medicine, Giresun, Turkey; d Department of Pathology, Giresun University Faculty of Medicine, Giresun, Turkey

Dear Editor,

We commend proffer our sincere commendation to Pitt and Haymart^1^ for their insightful commentary in thyroidology regarding the integration of tumor genetics into thyroid cancer staging. This study, progressing from the early detection of discrete genetic markers to the deployment of comprehensive commercialised panels employing DNA and RNA sequencing, presents challenges of singular import to our discipline. We readily agree with the authors that the signal work by Mingzhao Xing and his esteemed colleagues, who investigated these mutations across a multi-institutional, multinational cohort exceeding 4500 patients afflicted with Papillary Thyroid Carcinoma (PTC), represents a substantial stride toward informing future revisions of conventional guidelines. The study by Xing and colleagues underscores the prognostic value of coexisting BRAFV600E and TERTp mutations. A significant challenge remains the low prevalence of these markers: the overall proportion of patients with TERTp mutations is approximately 8.8%, while the co-existence rate of BRAF and TERTp is 5.9%. Furthermore, mutation rates show extreme variability, such as the 2.7% to 48.8% range observed in South Korean cohorts.^1^^,^^2^

Specifically, the identification that coexisting mutations improved prognostic accuracy across Stages I and II within the framework of the American Joint Committee on Cancer 8th Edition (AJCC8E) warrants profound attention. Notwithstanding these promising findings, we endorse the authors’ cautionary stance concerning widespread adoption, for several critical caveats remain.

Furthermore, the significant heterogeneity in the rate of mutation across various institutions and populations ‒ with the mutant rate spanning from 2.7% up to 48.8% in specific Korean cohorts ‒ raises profound queries regarding the optimal demographic for testing and the potential influence of environmental factors. Moreover, the incorporation of genetic profiles must be consonant with the contemporary movement toward the de-escalation of therapeutic interventions for low-risk papillary thyroid cancer, a malignancy frequently possessing an exceedingly favorable long-term prognosis. The authors themselves rightly stress the imperative of achieving a delicate “balance that improves prognostication without worsening overdiagnosis and overtreatment”.

Given that the AJCC8E currently stages all DTC similarly, while the cohort examined by Xing and colleagues solely investigated papillary thyroid cancer (excluding follicular and Hürthle cell carcinomas), the generalizability of these molecular findings is presently constrained. Of note, practical considerations pertaining to global, equitable implementation must not be overlooked; presently, the expense and general availability of testing impose palpable restrictions. The pathway forward necessitates a resolution regarding the optimal juncture for testing ‒ be it preoperatively or postoperatively ‒ and the precise mechanism for integrating it into risk-stratified surveillance.

As the future of staging evolves into a more personalised methodology, embracing traditional TNM stages with added molecular attributes, the precedent set by breast cancer staging within the AJCC8E ‒ which employs separate pathological and prognostic staging incorporating molecular data ‒ may prove instructive. We suggest that thyroidology^3^ should adopt a “prognostic staging” model similar to the AJCC8E model for breast cancer. This would integrate molecular data with clinico-pathological variables to prevent the overdiagnosis and overtreatment of low-risk patients. Ultimately, more comprehensive data are required to define an optimal, risk-stratified approach that might also consider additional attributes, such as sex or age, thereby ensuring that these advances yield clear public health benefits whilst avoiding unnecessary testing, increased treatment costs, and adverse psychological outcomes for patients. This issue merits further investigation. We extend our sincere appreciation to Drs. Pitt and Haymart^1^ for their scholarly work on tumour genetics and thyroid cancer staging for thyroidologists.

## Data availability

Data sharing is not applicable to this article as no new data were created in this study.

## Ethics approval

Not applicable.

## Artificial intelligence

No generative Artificial Intelligence (AI) or AI-assisted technologies have been used in the creation and production of this article.

## Authors’ contributions

IS: Conceptualization, formal analysis, ınvestigation, methodology, project administration, validation, visualization, writing-original draft, writing-review & editing.

DS: Conceptualization, formal analysis, ınvestigation, methodology, project administration, supervision, validation, visualization, writing-original draft, writing-review & editing.

## Funding

None declared.

## Conflicts of interest

The authors declare no conflicts of interest.

## References

[1] Pitt S.C., Haymart M. (2025). Tumour genetics and thyroid cancer staging: a balancing act. Lancet Oncol.

[2] Xing M., Lin S., Mathur A., Li Y., Kuklikova V., Melo M. (2025). Genetic modification of the AJCC classification of papillary thyroid cancer: an international, multicentre, retrospective cohort study. Lancet Oncol.

[3] Sengul I., Sengul D. (2026). A Consideration of the trends in thyroid fine-needle aspiration cytology – Results from the Italian Cytopathology Committee National Practice Survey in Thyroidology. Pathologica.

